# Genomic features of lung cancer patients in Indonesia’s national cancer center

**DOI:** 10.1186/s12890-024-02851-y

**Published:** 2024-01-20

**Authors:** Arif Riswahyudi Hanafi, Muhammad Alfin Hanif, Mariska T.G. Pangaribuan, Wily Pandu Ariawan, Noorwati Sutandyo, Sri Agustini Kurniawati, Lyana Setiawan, Dian Cahyanti, Farilaila Rayhani, Priscillia Imelda

**Affiliations:** 1 Department of Pulmonology, Dharmais Cancer Hospital, National Cancer Center, Letjen S. Parman Street Kav. 84-86 Slipi Jakarta Barat, DKI Jakarta, West Jakarta, 11420 Indonesia; 2 Department of Internal Medicine, Division of Hematology and Medical Oncology, Dharmais Cancer Hospital, National Cancer Center, West Jakarta, Indonesia; 3 Department of Clinical Pathology, Dharmais Cancer Hospital, National Cancer Center, West Jakarta, Indonesia; 4 Department of Anatomical Pathology, Dharmais Cancer Hospital, National Cancer Center, West Jakarta, Indonesia; 5 Cancer Research Team, Dharmais Cancer Hospital, National Cancer Center, West Jakarta, Indonesia

**Keywords:** Lung cancer, Molecular profile, Co-variant, ctDNA, NGS

## Abstract

**Introduction:**

Advances in molecular biology bring advantages to lung cancer management. Moreover, high-throughput molecular tests are currently useful for revealing genetic variations among lung cancer patients. We investigated the genomics profile of the lung cancer patients at the National Cancer Centre of Indonesia.

**Methods:**

A retrospective study enrolled 627 tissue biopsy samples using real time polymerase chain reaction (RT-PCR) and 80 circulating tumour DNA (ctDNA) liquid biopsy samples using next-generation sequencing (NGS) from lung cancer patients admitted to the Dharmais Cancer Hospital from January 2018 to December 2022. Data were obtained from medical records. Data statistically analysed with *p* < 0.05 is considered significant.

**Result:**

The *EGFR* test results revealed by RT-PCR were wild type (51.5%), single variant (38.8%), double variant (8.3%), and triple variant (1.4%), with 18.66% L85R, 18.22% Ex19del, and 11.08% L861Q variant. Liquid biopsy ctDNA using NGS showed only 2.5% *EGFR* wild type, 62.5% single variant and 35% co-variant, with *EGFR/TP53* and *EGFR/PIK3CA* as the highest.

**Conclusion:**

EGFR variants are the most found in our centre. Liquid biopsy with ctDNA using NGS examination could detect broad variants and co-variants that will influence the treatment planning.

## Introduction

The high burden of lung cancer is still a priority globally during the past few years. The latest global data showed that lung cancer is the second-highest cancer worldwide, estimating 2.2 million new cases per year [1]. The World Health Organization (WHO) summarized that lung cancer in Indonesia is the third most common cancer case, the leading cause of death due to cancer, and the most common cancer in males [2].

The advancement of molecular technology has an impactful value on cancer medicine. Several molecular tools have been developed to manage cancer efficiently. Thus, reducing the cancer incidence and mortality rate is now the target. Next-generation sequencing (NGS) is an emerging high-throughput technology in the field of molecular technology for DNA reading [3]. Since its development, NGS has been used by some hospitals worldwide since it led to a better understanding of cancer biology, including lung cancer. Using NGS techniques, broad genomic detection, including common and rare variants, are processed to find [4].

A recent study revealed that lung cancer in the Asian population is dominated by epidermal growth factor receptor (*EGFR*) variants, mainly for non-small cell lung carcinoma (NSCLC) [5]. The EGFR is a membrane-spanning glycoprotein featuring an external domain responsible for binding with epidermal growth factor and an internal tyrosine kinase domain, which governs cellular proliferation by controlling signal pathways. When EGFR binds with its ligand, it undergoes autophosphorylation through its intrinsic tyrosine kinase activity, initiating multiple signal transduction processes. Persistent or continual activation of these downstream pathways is believed to contribute to more aggressive tumour characteristics. Variants in *EGFR* have been identified in connection with certain types of lung cancers [6, 7]. Numerous resistance mechanisms have been documented in response to EGFR tyrosine kinase inhibitors (EGFR-TKIs). These include the emergence of secondary variants (such as T790M and C797S), the activation of alternative signalling pathways (involving Met, HGF, AXL, and IGF-1R), alterations in downstream pathways (e.g., AKT variants and loss of PTEN), disruptions in the EGFR-TKIs-induced apoptosis pathway (including BCL2-like 11/BIM deletion polymorphism), and histological transformations [8]. Other variants, such as Kirsten Rat Sarcoma virus (*KRAS*), tumour protein 53 (*TP53*), and phosphatidylinositol-4,5-bisphosphate 3-kinase catalytic subunit alpha (*PIK3CA*), also play a role in lung oncogenesis. Some of them have an essential impact on therapy and lung cancer prognosis [5].

Tissue biopsy is considered as the gold standard for *EGFR* variant test in lung cancer, mainly for NSCLC. However, circulating tumour DNA (ctDNA) from liquid biopsy is now considered rapid and accurate with reliable results since tumour access difficulty and intolerance to invasive procedures through tissue biopsy is often occurred [9]. The ctDNA is a short fragment of DNA shed by the tumour to the body fluid. It is representative of tumour characteristics, including lung cancer [10].

As a national cancer centre in Indonesia, there are estimated a thousand new people with lung cancer visiting us annually. We have been using real time polymerase chain reaction (RT-PCR) for several periods to detect some variants, and initially we used ctDNA with NGS technology to support lung cancer patients’ management, mainly for determining diagnosis and treatment. This study aims to investigate variant features of lung cancer patients at our centre, so it could provide a fundamental picture of lung cancer variants among the Indonesian population.

## Materials and methods

### Patients and data

A retrospective study enrolled 627 tissue biopsy samples for RT-PCR procedures and 80 ctDNA liquid biopsy samples for NGS procedures from patients diagnosed with lung cancer. Data were extracted from medical records from January 2018 to December 2022. Patients diagnosed with lung cancer through history taking and physical examination were directed to perform RT-PCR procedures or NGS procedures if they wanted to pay additional fees as the latest procedure is not fully covered by the government health insurance (BPJS). Furthermore, patients which were performed RT-PCR procedures but then had worsen clinical signs and symptoms after standard treatment, were suggested to perform the NGS procedure.

### Molecular procedures

Data of molecular studies were obtained from laboratory report. All molecular laboratory analyses were performed by KALGen Innolab, Jakarta, a cooperated laboratory with the Dharmais Cancer Centre, Indonesia.

#### Tissue biopsy with RT-PCR

- The slide tissue was used as the specimen type with tumour percentage of approximately 200 cells.
- FFPE tissue blocks were cut with a microtome and prepared into HE-stained and unstained tissue slides.
- Tumour areas were determined by pathologist using the HE stained slide. Then, the unstained slides were processed for deparaffinization using xylene and washing with 100% ethanol, 96% ethanol, 70% ethanol, and ddH_2_O, respectively.
- Marked tumour areas were scraped from the slide using sterile needle or blade and followed with DNA extraction using Qiagen QlAamp® DNA Microkit.
- The extraction process was performed according to the kit instruction. Briefly: tissue pellet was added with lysis buffer and proteinase K then incubated for 2 h. The entire lysate was transferred into the filtered column and centrifuged before washed with washing buffer 2 times. Then, the DNA was eluted with elution buffer.
- Variants in the EGFR gene were analysed using PCR based on High Resolution Melting (HRM) PCR as the rapid screening process.
- Undetected variants from the process were scored and considered as wild type.
- The detected variants were confirmed with fragment analysis or direct sequencing with Sanger Method.
- Results shown as positive variants which covered Exon 18 G719/A/C/S/D, Ins Exon 19, Del Exon 19, Del Exon 18, Exon 20 T790M, Exon 20 S7681, Exon 21 L858R, and Exon 21 L861Q.

#### Liquid biopsy with NGS

- Serum from whole blood was used as the specimen.
- Circulating tumor DNA/ctDNA was extracted from sample using the MagMAX cell free Total Nucleic Acid (MagMAX cfTNA) protocol.
- DNA concentration was quantified using NanoDrop spectrophotometer (Nanodrop Technologies, Wilmington, DE, USA) and a Qubit Fluorometer (Invitrogen, Carlsbad, CA, USA).
- The DNA Library was prepared wtih Oncomine™ Lung cfDNA Assay (Thermo Fisher Scientific).
- This panel includes 12 different genes covering > 169 hotspots, 49 fusions, and MET exon 14 skipping.
- Twelve genes covered are as follows: ALK, KRAS, PIK3CA, BRAF, MAP2K1, RET, EGFR, MET, ROS1, ERBB2, NRAS, TP53.
- Prepared DNA library was sequenced using Next Generation Sequencing (NGS) Ion GeneStudio™ S5 System and data analysis was performed with the Ion ReporterTM 5.10.1.0 software directly from within Torrent SuiteTM 5.10.1 software (Thermo Fisher Scientific), followed by manual inspection.
- In the variant details, the software analysis will only show positive results while negative results will be filtered out automatically.

### Data analysis

The data were analysed using IBM SPSS Statistics (version 25.0; IBM Corp, NY). Data are presented in frequency and percentages. Data were descriptively compared between variables and assessed using the *chi-square* test, with a p-value (p) < 0.05 considered statistically significant.

## Results

There were 707 subjects included in this study. Basic demographic information is described in Table 1.

**Table 1:** Basic demographic information

Demographic variables	Frequency (n)	Percentage (%)
**Age**		
Adult (< 40 y)	40	5.6
Middle-aged (40–60 y)	361	51.1
Elderly (> 60 y)	306	43.3
**Gender**		
Male	454	64.2
Female	253	35.8
**Smoking history**		
Smoker	371	52.5
Non-smoker	336	47.5
**Lung cancer type**		
Adenocarcinoma	681	96.3
Adeno-squamous carcinoma (ASC)	1	0.1
Squamous cell carcinoma (SCC)	22	3.2
Large cell carcinoma	1	0.1
Small cell lung carcinoma (SCLC)	2	0.3

From Table 1, there were about 43.3% of subjects aged above 60 years and 51.1% between 40 and 60 years. Meanwhile, the rest were under 40 years old. More than half of the total subjects were male (64.2%). Smoker subjects were slightly dominant (52.5%) compared to non-smokers (47.5%). Most lung cancer types were adenocarcinoma (96.3%), followed by squamous cell carcinoma (3.2%), small cell lung carcinoma (0.3), and small portion of other types, such as adeno-squamous carcinoma (0.1%) and large cell carcinoma (0.1%).

A total of 627 samples analysed with EGFR RT-PCR are shown in Table 2. Most patients represented wild type (51.5%). Single variants constituted 38.8%, while double variants were about 8.3%. There was only a tiny number of *EGFR* triple variants expressed among subjects (1.4%).

**Table 2:** Genomic profile from tissue biopsy using EGFR RT-PCR

EGFR Test	Frequency (n)	Percentages (%)
Wild type	323	51.5
Single variant	243	38.8
Double variant	52	8.3
Triple variant	9	1.4

EGFR, epidermal growth factor receptor

Table 3 illustrates the number of *EGFR* variants among lung cancer types detected by RT-PCR. Adenocarcinoma was mostly constituted by wild types (50.8%), followed by single variants of Ex19del (15.9%), L858R (14.8%), and L861Q (5.8%), double variant of L858R/L861Q (3.3%), and a small number of other variants. Meanwhile, in squamous cell carcinoma (SCC), more than 50% patients were wild type, others were single variant constituted by Ex19del (13.9%), L816Q (9%), and same number of L858R and G719 A/C/S/D (4.5%), with none of this group showing both double and triple variants. Other NSCLC subjects expressed only wild type and a single variant of L858R (50%). In addition, one patient with SCLC showed wild-type result after the test (100%).

**Table 3:** The results among lung cancer types detected by EGFR RT-PCR

Result		n (%)				
		Adenocarcinoma	SCC	Other NSCLC	SCLC	Total
Wild type		306 (50.8%)	15 (68.2%)	1 (50%)	1 (100%)	323
Single variant	L858R	89 (14.8%)	1 (4.5%)	1 (50%)	0	243
	L861Q	35 (5.8%)	2 (9.0%)	0	0	
	Ex19del	94 (15.6%)	3 (13.6%)	0	0	
	G719 A/C/S/D	15 (2.5%)	1 (4.5%)	0	0	
	G718 S	1 (0.2%)	0	0	0	
	Ex20ins	1 (0.2%)	0	0	0	
Double variant	Ex19del/L858R	10 (1.6%)	0	0	0	52
	Ex19del/L861Q	12 (2.0%)	0	0	0	
	L858R/L861Q	20 (3.3%)	0	0	0	
	L861Q/G719 A/C/S/D	3 (0.5%)	0	0	0	
	L858R/G719 A/C/S/D	1 (0.2%)	0	0	0	
	Ex19del/G718 A/C/S/D	2 (0.3%)	0	0	0	
	Ex19del/T790M	2 (0.3%)	0	0	0	
	L858R/T790M	1 (0.2%)	0	0	0	
	Ex19del/Ex18del	1 (0.2%)	0	0	0	
Triple variant	L858R/L861Q/G719A	3 (0.5%)	0	0	0	9
	Ex19del/L858R/L861Q	4 (0.6%)	0	0	0	
	Ex18del/L858R/L861Q	1 (0.2%)	0	0	0	
	Ex19del/L861Q/G719S	1 (0.2%)	0	0	0	
Total		602	22	2	1	627

SCC, squamous cell carcinoma; NSCLC, non-small cell lung carcinoma; SCLC, small cell lung carcinoma

**Fig. 1:**
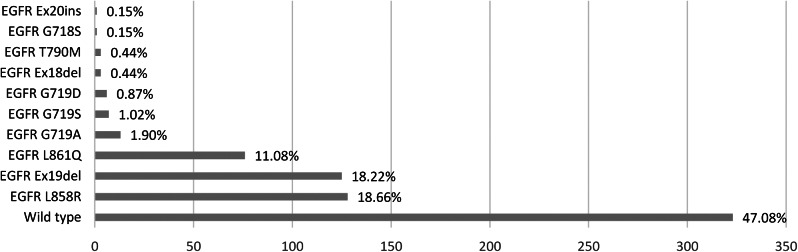
The breakdown of each EGFR variant detected by RT-PCR

From Fig. 1, around 47.08% of the total results were wild type. The L858R and Ex19del variants had quite similar numbers, 18.66% and 18.22%, respectively. Meanwhile, approximately 11.08% are L861Q variant. A few other variants were also expressed, such as G719, Ex18del, T790M, G718S, and Ex20ins (< 2.00%).

In Table 4, we grouped the genomic landscape of NGS results into three categories, which are single gene variant, co-variant, and wild type. Co-variant was defined as two gene variants detected in one subject. From a total of 80 subjects, there were 62.5% of subjects having single gene variants, 35% with co-variants, and only two patients had wild type lung cancer (2.5%).

**Table 4:** Genomic profile from ctDNA liquid biopsy using NGS

Variant	Frequency (n)	Percentage (%)
Single gene variant	50	62.5%
Co-variant	28	35%
Wild type	2	2.5%
TOTAL	80	

EGFR, epidermal growth factor receptor; TP53, tumour protein 53; KRAS, Kirsten Rat Sarcoma virus; PIK3CA, phosphatidylinositol-4,5-bisphosphate 3-kinase catalytic subunit alpha; BRAF, B-Raf proto-oncogene; MET, mesenchymal epithelial transition; ROS1, reactive oxygen species 1; MAP2K1 mitogen activated protein kinase 1; NRAS (neuroblastoma RAS viral (v-ras) oncogene homolog.

Table 5 shows each variant from NGS test among lung cancer types. From 80 subjects included, only one patient was diagnosed with SCC and had *EGFR* variant, while the rest was diagnosed with adenocarcinoma. Of the patients with adenocarcinoma, 53.2% had *EGFR* single gene variants, followed by 6.2% with *TP53*, and a small portion had *KRAS* and *PIK3CA* single gene variants (1.3% each). Meanwhile, the co-variant detected was mostly *EGFR* with other gene(s), with *EGFR/TP53* being the prominent one (13.9%), followed by *EGFR/PIK3CA* (8.9%), *EGFR/MET* (2.4%), and a few other *EGFR* co-variants (1.3% each). There were two subjects having rare co-variant, namely *KRAS/TP53* and *NRAS/TP53*. In addition, only two subjects were wild type (2.4%).

**Table 5:** The results among lung cancer types detected by NGS

Result		n (%)				
		Adenocarcinoma	SCC	Other NSCLC	SCLC	p-value
Single gene variant	EGFR	42 (53.2%)	1 (100%)	0	0	0.000
	TP53	5 (6.2%)	0	0	0	
	KRAS	1 (1.3%)	0	0	0	
	PIK3CA	1 (1.3%)	0	0	0	
Co-variant	EGFR/TP53	11 (13.9%)	0	0	0	0.000
	EGFR/TP53/KRAS	1 (1.3%)	0	0	0	
	EGFR/TP53/KRAS/PIK3CA	1 (1.3%)	0	0	0	
	EGFR/KRAS	1 (1.3%)	0	0	0	
	EGFR/KRAS/BRAF	1 (1.3%)	0	0	0	
	EGFR/MET	2 (2.4%)	0	0	0	
	EGFR/PIK3CA	7 (8.9%)	0	0	0	
	EGFR/PIK3CA/MAP2K1	1 (1.3%)	0	0	0	
	EGFR/ROS1	1 (1.3%)	0	0	0	
	KRAS/TP53	1 (1.3%)	0	0	0	
	NRAS/TP53	1 (1.3%)	0	0	0	
Wild type		2 (2.4%)	0	0	0	0.000
**Total**		79	1	0	0	

EGFR, epidermal growth factor receptor; TP53, tumour protein 53; KRAS, Kirsten Rat Sarcoma virus; PIK3CA, phosphatidylinositol-4,5-bisphosphate 3-kinase catalytic subunit alpha; BRAF, B-Raf proto-oncogene; MET, mesenchymal epithelial transition; ROS1, reactive oxygen species 1; MAP2K1 mitogen activated protein kinase 1; NRAS (neuroblastoma RAS viral (v-ras) oncogene homolog

**Fig. 2:**
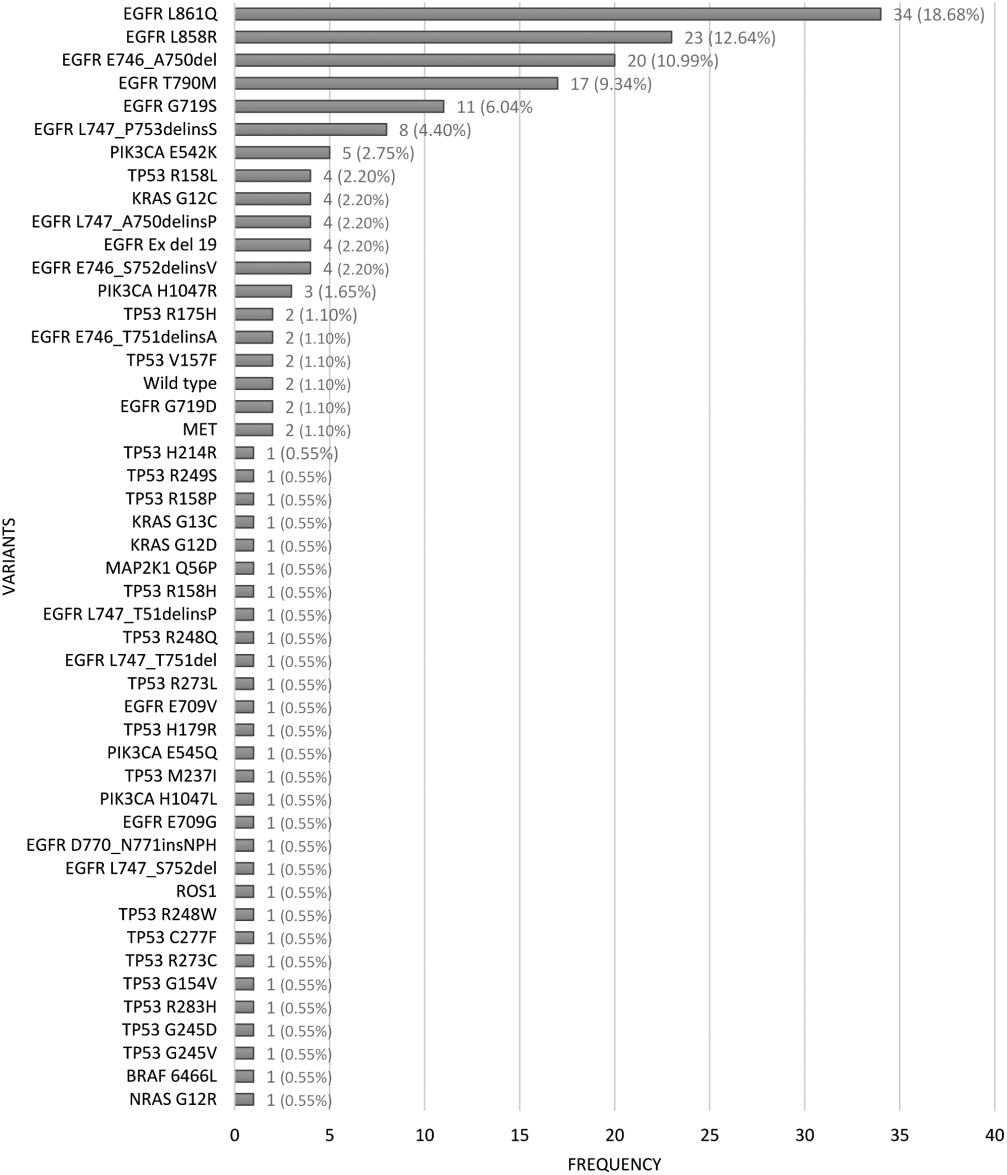
The breakdown of each variant expressed from NGS

Each variant from NGS result is illustrated in Fig. 2. The *EGFR* L861Q was most frequently appeared in lung cancer patients (18.68%). Meanwhile, *EGFR* L858R constituted the second highest (12.64%). *EGFR* E746_A750del, T790M, G719S, and L747_P753delinsS were the next most expressed among patients in our centre. Additionally, there was a small count of other variants from other genes, such as *TP53, PIK3CA, KRAS, NRAS, BRAF, ROS1, MAP2K1*, and *MET.*

## Discussion

The incidence of lung cancer is one of the highest among cancers worldwide according to data from the International Agency for Research of Cancer (IARC) [1]. This study found that people with lung cancer were dominated by middle-aged (40–60 years) group and elderly (above 60 years), with a ratio between males and females of approximately 2:1. As one of the risk factors, both smoking exposure and non-smoking contribute to lung cancer pathogenesis, and in this study, we found slight differences between both. Epidemiologically, about 85% of lung cancer type is non-small cell lung cancer (NSCLC), with the rest being small cell lung carcinoma (SCLC) [11, 12]. This study revealed that NSCLC was the highest among other types, consisting of mostly adenocarcinoma.

Several current guidelines recommend that molecular tests should be considered in tailoring the treatment due to their high sensitivity and specificity, including for lung cancer [13]. In the lung cancer, most molecular test considerations are mainly due to their link to the treatment option, such as tyrosine kinase inhibitor (TKI) [14]. Furthermore, the partnership between the College of American Pathologists (CAP), the International Association for the Study of Lung Cancer (IASLC), and the Association for Molecular Pathology (AMP) has released the molecular testing guideline for treatment with TKI in lung cancer patients [15]. Thus, the investigation of molecular biology is now becoming an issue.

We have applied molecular tests in our services, especially for lung cancer management. We considered that *EGFR* variants must be the main concern since it is epidemiologically common in the Asian population (30–40%) compared to the European population (10–15%) [16]. Through this study, we found significant differences regarding the number of variants found using RT-PCR and NGS, especially for *EGFR* variant.

It is important to note that the number of samples tested using RT-PCR were far more than using NGS (627 samples vs. 80 samples, respectively). However, there is a reasonable explanation for this finding. Most patients in our centre use the national health insurance that only cover the RT-PCR test. Thus, the NGS test is only optional as it needs additional payment which were not suitable for all patients. However, for patients which had performed RT-PCR and had received treatment but their cancer relapse or unresponsive after treatment, we usually recommend them other tests, such as NGS. Therefore, it is also possible that the characteristics of patients which were performed NGS might be with higher resistance to treatment population than the RT-PCR. This could explain the higher *EGFR* T790M variant ratio in NGS cohort (Fig. 2) compared with the PCR cohort (Fig. 1). This difference is not a vital as the objective of this study was to depict the variant profile of lung cancer patients in our centre, not to compare RT-PCR and NGS as it has been studied before by Kosasih et al. [17]. It reported that NGS is more specific to detect *EGFR* variant than PCR. For some patients which were assessed as wild type by PCR examination, the results from NGS might detect more specific variant, such as *EGFR* E746_A750del, L858R, or L861Q variant [17].

Variants in *EGFR* may lead to cancer signalling pathways, such as in lung cancer [18, 19]. From this study, half of the subjects were *EGFR* wild-type lung cancer detected by RT-PCR. However, there were a portion of single variants, double variants, and triple variants. EGFR variant is divided into common or “classical” variants and uncommon or rare variants, with their implication on both predictive and prognostic value [20]. EGFR variants mainly occur within exons 18 to 21, with approximately 85–90% of them being common variants and the rest being uncommon. The most common variant is exon 19 deletions (Ex19del) and L858R variant in exon 21. Meanwhile, *EGFR* G719X, L861Q, Ex20ins, and S7681 variant are the major uncommon variants. Additionally, EGFR T790M and E746_A750del are considered less frequent uncommon variants [20, 21]. A current study said that *EGFR* alterations in NSCLC patients would be a candidate for targeted TKI therapy [14]. Several studies have shown that variants involving exons 18, 19, and 21 are considered sensitive to EGFR-TKI therapy, whereas those involving exon 20 variants are typically less sensitive or resistant [20–23]. Among Asian ethnicities, Ex19del and L858R variants were the most common [24, 25]. Another study has summarized molecular epidemiology of *EGFR* variations among advanced NSCLC patients in 7 Asian countries, excluding Indonesia. It showed that most of variants were L858R and Ex19del, while L861Q variants implied in not more than twenty patients, both in the form of single and combination variants [26]. Confirming this epidemiological data, this study with RT-PCR test showed that Ex19del and L858R variant were dominated compared with others, while many other subjects possessed double and triple variants, which were a combination of common and rare variants. Combination with uncommon variants, such as L861Q, G719X, T790M, and Ex18del, were detected by RT-PCR in this study. Thus, it could be challenging for clinicians in tailoring further therapy and estimating the prognosis.

Several patients were followed up to determine other variants that might affect the treatment response. We could highlight that RT-PCR investigation is limited to the certain gene. Considering liquid biopsy using NGS has become a good choice since it can detect variants within multiple genes by designing gene panels. Some copy number variations might be also detected, which are important for deciding the treatment. We did not compare the results between *EGFR* variant from RT-PCR and from NGS because it has been done by other researchers at our centre. However, we included the NGS results as we want to picture a broad range of variants using NGS that might be beneficial to add some basic epidemiological data, both globally and nationally.

Although most patients had a single gene variant detected by NGS, there were considerably 35% of patients with co-variant, which was dominated by *EGFR* compounding variant with other gene(s). During the past few years, many global studies have focused on these co-variant and their effect on TKI-based treatment [14, 19, 27]. Epidemiologically, *EGFR* variants are abundantly expressed among Asian populations. Recent Asian expert consensus has reported that the percentage of *EGFR* alterations in lung adenocarcinoma is higher among East Asian compared to Western population (40–55% vs. 12–25%), while *KRAS, BRAF*, and *ROS1* are lower, with *HER2* and *MET* are nearly similar [28]. However, coinciding with other genes will influence patients’ respond toward treatment. This study indicated that *EGFR/TP53* co-variant and *EGFR/PIK3CA* co-variant were largely expressed among lung cancer patients, knowing both *TP53* and *PIK3CA* variants are important for survival prognosis in lung cancer [9].

The *TP53* gene is a tumour suppressor gene, mainly encoding tumour protein p53, a transcription factor essential for cell cycle control, especially for DNA repair and cell apoptosis [29]. According to several prior studies, approximately 50% of their subjects with lung cancer had *TP53* variants [30, 31]. A previous study showed that patients with *EGFR*-mutated NSCLC with concomitant *TP53* variant were associated with a poorer clinical prognosis [32]. Additionally, a study in the Brazilian population concluded that concurrent variant of *TP53* had a potentially negative predictive effect associated with platinum-based chemotherapy and erlotinib in early-stage EGFR-mutated NSCLC [33]. A Meta-analysis research also stated that *TP53* variants represent a clinically relevant mechanism of resistance to EGFR-TKIs, regardless of their generation [34]. Moreover, a multi-omics cohort of East Asian population has reported that *EGFR/TP53* co-variant display distinct biological features and has worse prognosis than EGFR single variant [35]. However, the best strategy in choosing treatment for this co-variant is little known. Some investigations have been developed focusing on the p53 target gene for upcoming treatment, but the drug at present is still unavailable [32]. Thus, a remarkable number of *TP53* co-variants among the Indonesian population in our cohorts should be beneficial in estimating future treatment planning.

In addition, a concomitant *PIK3CA* variant should be considered as a poor prognosis. The *PIK3CA* is a tumour-specific gene that encodes type IA P13K protein to activate the P13K/Akt/mTOR signalling pathway in promoting cell proliferation [36]. Variant in this gene will increase lipid kinase activity and the Akt signalling pathway that leads to tumours, including lung cancer [37]. A study showed that NSCLC patients with *EGFR* variants coexisting with *PIK3CA* variant (co-variant) had a decrease in median overall survival compared with a single gene *EGFR* variant [38].

Another variant in lung cancer cases was *KRAS* variant. The *KRAS* gene is one of the other three human RAS families that encodes GTPase KRAS protein. This protein is responsible for cell proliferation, differentiation, and survival [39]. A previous study stated that *KRAS* variant is quite frequent in adenocarcinoma and squamous cell lung carcinoma [40]. Alteration in *KRAS* is implicated in lung cancer pathogenesis, mainly for patient survival. A study showed that lower expression on RAS was associated with longer survival time [41]. A recent study on genomic landscape among East Asian population showed that although 48.6% of samples had *EGFR* driver variants, co-variants of *EGFR* and *KRAS* still occurred in 4.7% of samples [42]. This study showed that some patients had *KRAS* variant, both in single variant and co-variant, which needs to be further evaluated.

There were other concurrent variants, such as, *NRAS, BRAF, MP2KI, ROS1*, and copy number variation on *MET*. However, they were in small numbers. We supposed that because our sample size was limited. However, something interesting was found in this study. There were triple and even quadruple co-variants, such as *EGFR/TP53/KRAS/PIK3CA* co-variant. This is a novel finding since no other related study has discovered or discussed triple or quadruple co-variant in lung cancer patients as we had. We cannot elaborate more about the mechanism affecting lung cancer, but we suppose that the number of co-variants might worsen patients’ condition, especially in achieving treatment efficacy. A study in Chinese discussed *EGFR-BRAF* co-variant, but without *KRAS* variant [43]. Another study also found *MAPK1* variant, a rare variant in lung cancer, but no other evidence found co-variant with EGFR and PIK3CA [44]. A previous study discovered some concurrent genomics alteration related to EGFR-TKI resistance. It stated that *KRAS, ROS1*, and *MET* (both polysomy, and amplification) were included in the group of concurrent driver gene alteration [45]. In addition, this study revealed coexisting variant between *NRAS* and *TP53.* No other study has reported this co-variant in lung cancer. The *NRAS* gene is one of the RAS proteins like KRAS which encode GTPase NRas protein to promote cell proliferation [46]. It is extremely rare among lung cancers. A study in Japan reported that only 1 of 195 cases of *NRAS* variant and was considered a somatic variant in lung cancer patients [47].

We described each variant from NGS test specifically in Fig. 2 to be easily visualized. The most common variants detected by NGS were dominated by *EGFR* driver gene variants, with top 5 among all variants were all variations within *EGFR* gene, which are L861Q, L858R, E746_A750del, T790M, and G719S. We only highlight the top 5 among all and will not elaborate on all variants revealed in this study because there were too many. These five variants are mostly rare variants of *EGFR*. Globally, *EGFR* rare variants are abundantly detected, especially with high-throughput investigation. From NGS examination, it could be highlighted that *EGFR* rare variants seem to be more dominant compared with RT-PCR results (Fig. 2).

The L861Q variant was the most detected among other *EGFR* rare variants (Fig. 2). It is found on exon 21 of the *EGFR*, a tyrosine kinase domain. A previous study in China also found that L861Q variant was a frequent feature of NSCLC among their population, and its sensitivity to the treatment has been studied [27]. Furthermore, another study in Tunisia also reported that L861Q variant have been found in 35.3% (12 out of 34 cases), ranking it as the highest rare variant found among patients with non-small cell lung cancer [48]. However, there are also other studies that reported L861Q variant as a very rarely found variant among their population. For example, study by Mistudomi et al. in Japan confirmed that they only found 2% of this variant among lung cancer patients [49]. In addition, a case report by Hines et al. in the United States also reported that L861Q variant was a very rare case in the USA [50]. According to the latest research so far, the clinical significance of L861Q variant in lung cancer patients is still undiscovered well. However, there are several studies that pointed out the effect of this variant to the sensitivity to treatment. Some preclinical studies have shown that the L861Q variant may exhibit limited effectiveness or complete resistance to EGFR tyrosine kinase inhibitors (EGFR-TKIs), implying a grim outlook for these patients [51, 52]. However, a significant retrospective study conducted by Chiu et al. reported a response rate (RR) of 39.6% and a progression-free survival (PFS) of 8.1 months, indicating a moderate response to TKIs. Unfortunately, the overall survival (OS) was not tracked in this study [22]. Wu and Xu also obtained similar findings to Chiu, albeit with a smaller sample size [53]. In a prospective clinical trial led by Yang, high afatinib activity was observed in patients carrying the L861Q variant, with an objective response rate (ORR) of 56.3%, a median PFS of 8.2 months, and a median OS of 17.1 months [54].

Other *EGFR* rare variants, such as E746_A750del, T790M, and G719S, were also found by NGS and were dominated in the top 5 (Fig. 2). Besides, there were also a significant number of L747_P753delinsS and Ex19delins, which could not be revealed by RT-PCR test. Several studies concentrated *EGFR* exon 19 variations of lung cancer patients among Chinese population. A study using targeted panel sequencing revealed no *EGFR* variants of E746_A750del and L747_P753delinsS [55]. Meanwhile, another study with sanger sequencing showed 64.6% of E746_A750del variant and detected no L747_P753delinsS variant [56]. Multi-centre study using NGS reported 24 cases of L747_P753delinsS variant [57]. A E746_A750del variant causes the loss of intratumoral CD8 + cells, which can repress the antitumor immunity system [58]. However, some studies reported the contrary outcomes of *EGFR* sub’ response upon TKI-therapy [59, 60]. Moreover, a prior study has shown that although Ex19delins has a better survival outcome than Ex19del variant, the acquisition of T790 variant in *EGFR* Ex19delins group would make them have a poorer prognosis [61]. Additionally, one patient also expressed Ex20ins variant in this study, which is linked to less sensitive therapy and less favourable outcomes compared with other uncommon variants. As we stated before, this challenging profile should be addressed well, and it needs more database to look up for choosing the effective treatment.

We realized that this study did not further explain all variants stated, including some rare variants revealed by NGS. This study focuses on descriptively explaining the genomic profile of variants in several genes and their frequencies. Besides, our sample size for NGS was relatively small. This is probably due to the NGS application in our centre initially beginning in 2019, so it affects the overall number of patients undergoing NGS procedures. However, we know that examination with NGS detects more extended variants and co-variant than RT-PCR, and it should be considered since several co-variants, such as *EGFR/TP53, EGFR/PIK3CA*, and *EGFR/KRAS*, would affect treatment response. We expect that this study could provide fundamental data for others in the future, especially for genomic landscape of lung cancer among Asian populations. We also recommend that other studies with a larger sample size should be further conducted.

## Conclusion

EGFR variants were highly expressed among lung cancer patients. Liquid biopsy ctDNA using NGS examination showed extended variant and co-variants that will impact treatment designing.

## Data Availability

Final datasets are available on https://datadryad.org/stash/share/jB5vDmMlYIUnR-4XP7z-2hpKh6HR1vp99vft1sUHhvg. The genomic dataset used for this research are secondary data from third-party company (a cooperated laboratory). The raw data cannot be shared publicly for privacy reasons. The data are however available from the corresponding author with reasonable request.

## List of Abbreviations

WHO: World Health Organization
NGS: Next-generation sequencing
EGFR: Epidermal growth factor receptor
NSCLC: Non-small cell lung carcinoma
KRAS: Kirsten Rat Sarcoma virus
TP53: Tumor protein 53
PIK3CA: Phosphatidylinositol-4,5-bisphosphate 3-kinase catalytic subunit alpha
ctDNA: Circulating tumor DNA
RT-PCR: Real time polymerase chain reaction
TKI: Tyrosine kinase inhibitor
CAP: College of American Pathologists
IASLC: International Association for the Study of Lung Cancer
AMP: Association for Molecular Pathology
BRAF: B-Raf proto-oncogene
MET: mesenchymal epithelial transition
ROS1: reactive oxygen species 1
MAP2K1: mitogen activated protein kinase 1
NRAS: neuroblastoma RAS viral (v-ras) oncogene homolog
